# High GILT Expression Is Associated with Improved Survival in Metastatic Melanoma Patients Treated with Immune Checkpoint Inhibition

**DOI:** 10.3390/cancers14092200

**Published:** 2022-04-28

**Authors:** Anngela C. Adams, Elizabeth S. Borden, Anne M. Macy, Nick Thomson, Haiyan Cui, Mark I. Gimbel, Melissa A. Wilson, Kenneth H. Buetow, Denise J. Roe, David J. DiCaudo, Jade Homsi, Karen Taraszka Hastings

**Affiliations:** 1 College of Medicine-Phoenix, University of Arizona, Phoenix, AZ 85004, USA; anngelaa@email.arizona.edu (A.C.A.); knodele@email.arizona.edu (E.S.B.); amacy@email.arizona.edu (A.M.M.); 2 Phoenix Veterans Affairs Health Care System, Phoenix, AZ 85012, USA; 3 Banner MD Anderson Cancer Center, Gilbert, AZ 85234, USA; nick.Thomson@RevereHealth.com (N.T.); mark.gimbel@bannerhealth.com (M.I.G.); jade.homsi@UTSouthwestern.edu (J.H.); 4 University of Arizona Cancer Center, University of Arizona, Tucson, AZ 85719, USA; hcui@uacc.arizona.edu (H.C.); droe@arizona.edu (D.J.R.); 5 School of Life Sciences, Arizona State University, Tempe, AZ 85281, USA; mwilsons@asu.edu (M.A.W.); kenneth.buetow@asu.edu (K.H.B.); 6 Center for Evolution and Medicine, Arizona State University, Tempe, AZ 85281, USA; 7 Mel and Enid Zuckerman College of Public Health, University of Arizona, Tucson, AZ 85724, USA; 8 Department of Dermatology, Mayo Clinic, Scottsdale, AZ 85259, USA; dicaudo.david@mayo.edu

**Keywords:** metastatic melanoma, GILT, immune checkpoint inhibitors

## Abstract

**Simple Summary:**

Skin cancer is the most common type of cancer, with melanoma being among the deadliest of skin cancers due to its propensity to metastasize. Immune checkpoint inhibitors (ICI) generate anti-tumor immune responses resulting in improved outcomes in patients with metastatic melanoma. However, only a subset of melanoma patients responds to these therapies, which are costly and come with a risk of adverse effects. Therefore, there is a need for biomarkers to predict which patients will respond to ICI. We found that ICI-treated metastatic melanoma patients with high GILT mRNA expression in bulk tumor samples had improved survival. Additionally, high GILT protein expression within metastatic melanoma cells was associated with improved survival in patients treated with ICI. This study suggests that GILT may serve as a biomarker to predict which patients will respond to ICI, which could improve patient care, reduce healthcare costs, and facilitate appropriate selection of therapies for patients with metastatic melanoma.

**Abstract:**

Gamma-interferon-inducible lysosomal thiol reductase (GILT) is critical for MHC class II restricted presentation of multiple melanoma antigens. There is variable GILT protein expression in malignant melanocytes in melanoma specimens. High GILT mRNA expression in melanoma specimens is associated with improved overall survival, before the advent of immune checkpoint inhibitors (ICI). However, the association of GILT in metastatic melanoma with survival in patients treated with ICI and the cell type expressing GILT associated with survival have not been determined. Using RNA sequencing datasets, high GILT mRNA expression in metastatic melanoma specimens was associated with improved progression-free and overall survival in patients treated with ICI. A clinical dataset of metastatic melanoma specimens was generated and annotated with clinical information. Positive GILT immunohistochemical staining in antigen presenting cells and melanoma cells was observed in 100% and 65% of metastatic melanoma specimens, respectively. In the subset of patients treated with ICI in the clinical dataset, high GILT protein expression within melanoma cells was associated with improved overall survival. The association of GILT mRNA and protein expression with survival was independent of cancer stage. These studies support that high GILT mRNA expression in bulk tumor samples and high GILT protein expression in melanoma cells is associated with improved survival in ICI-treated patients. These findings support further investigation of GILT as a biomarker to predict the response to ICI.

## 1. Introduction

Melanoma is a common skin cancer with the propensity to metastasize to lymph nodes and distant organs. The American Cancer Society estimates that 99,780 new cases of melanoma will be diagnosed and 7650 people will die from melanoma in 2022 in the United States [1]. Immune checkpoint inhibitors (ICI), which block the inhibitory receptors PD-1 and CTLA-4 on T cells, have transformed the treatment of advanced melanoma. However, only 33–44% of melanoma patients respond to monoclonal antibodies (mAb) against PD-1 [2,3,4], and 58% of melanoma patients respond to combination therapy with mAb targeting PD-1 and CTLA-4 [2]. In addition, ICI are costly and come with a risk of adverse events. Thus, there is an increasing need for biomarkers to assist clinical decision making in selecting which patients are most likely to respond to ICI.

CD4 and CD8 T cells have a dual role in effective anti-tumor responses. CD8 T cells have a direct role in killing MHC class I-expressing tumor cells. The role of CD8 T cells in tumor destruction is highlighted by the loss of MHC class I or components of the MHC class I pathway as mechanisms of immune evasion and resistance to cancer treatment with ICI [5,6,7,8,9]. CD4 T cell responses, which are activated via the MHC class II antigen presentation pathway, are required for optimal primary CD8 T cell responses and formation of memory CD8 T cells, through activation of antigen presenting cells (APCs) and secretion of cytokines [10,11]. CD4 T cells play essential roles in anti-tumor immune responses by enhancing the influx, efficacy, and duration of CD8 T cell responses [12,13,14]. Additionally, CD4 T cells can exhibit direct cytotoxicity of tumor cells [15,16,17,18] and indirectly kill tumor cells through activation of APCs [18]. In a murine melanoma model, adoptive cell therapy with CD4 and CD8 T cells results in enhanced tumor regression compared to adoptive transfer of CD8 T cells alone [19]. In murine melanoma and colon adenocarcinoma models, both CD4 and CD8 T cells are necessary for response to anti-PD-1 treatment [20]. In a murine sarcoma model, response to ICI requires dual expression of both an MHC class I- and MHC class II-restricted tumor-specific antigen in tumor cells [21]. Yet, the role of the MHC class II pathway within the tumor microenvironment and in tumor cells remains to be fully understood.

The MHC class II antigen presentation pathway generates peptide–MHC class II complexes for the activation of CD4 T cells. Gamma-interferon-inducible lysosomal thiol reductase (GILT), a component of the MHC class II pathway, is encoded by the IFI30 gene and is an enzyme localized to the endocytic compartment where MHC class II loading occurs [22]. GILT catalyzes the reduction of protein disulfide bonds, and this enzymatic activity facilitates the presentation of certain MHC class II-restricted peptide epitopes [23], likely through exposing buried epitopes for MHC class II binding. GILT is required for efficient MHC class II-restricted presentation of melanoma antigens, including tyrosinase and tyrosinase-related protein 1, by both APCs and melanoma cells [24,25,26]. GILT expression accelerates the onset and intensity of CD4 T cell responses in vivo [25]. While expression of the MHC class II antigen presentation pathway, including GILT, is generally limited to professional APCs, GILT can be constitutively expressed or induced in tumor cells, such as melanoma [24,27,28,29,30]. GILT expression in melanoma cells is induced by interferon-γ (IFN-γ) and tumor necrosis factor-α (TNF-α), and to a lesser extent by interleukin-1β (IL-1β) [27]. Whereas GILT and MHC class II proteins are not detected in melanocytes in benign, uninflamed nevi specimens, GILT and MHC class II protein expression is detected in melanoma cells in 60% and 30% of metastatic melanoma specimens [31], respectively. In contrast, GILT-expressing, MHC class II-expressing APCs are present in all melanoma specimens [31]. GILT protein is expressed within melanocytes of halo nevi, which are regressing nevi with a brisk lymphocytic infiltrate known to be enriched in TNF-α [32]. The variation of GILT expression in melanoma cells and induction of GILT in melanocytes of regressing nevi suggest that GILT expression in melanoma cells may be associated with improved clinical outcome.

An increasing number of studies have shown an association of high GILT expression in human cancer specimens with improved clinical outcome. High GILT mRNA expression was first shown to be associated with improved survival in diffuse large B cell lymphoma [30]. Subsequently, GILT, either as an independent gene or as a gene signature, has been identified to be positively associated with survival in adenocarcinoma at the gastroesophageal junction and other chromosomal instability-type gastric cancers [33], clear cell renal carcinoma [34], and breast cancer [35,36], and is associated with response to therapy in acute myeloid leukemia [37]. In cutaneous melanoma specimens prior to the advent of ICI, high GILT mRNA expression is associated with improved survival [27]. Here, we report the association of GILT expression with melanoma with response to ICI, using GILT mRNA expression in bulk tumor specimens and GILT protein expression in melanoma cells by immunohistochemistry.

## 2. Materials and Methods

### 2.1. Data Sets

RNA sequencing (RNAseq) data were obtained for the Liu et al. and Van Allen et al. datasets from the Melanoma Genome Sequencing Project (dbGaP accession number phs000452.v3.p1) [38,39]. RNAseq data were obtained for the Hugo et al. dataset from the gene expression omnibus repository with accession number GSE78220 [40]. The Liu et al. dataset consisted of 122 melanoma samples, the Van Allen et al. dataset consisted of 36 melanoma samples, and the Hugo et al. dataset consisted of 26 melanoma samples. Sex distributions, melanoma subtypes, stage, and treatments are summarized in Table 1. All RNAseq data were obtained as raw FASTQ read files.

### 2.2. Transcriptome Data Preparation

RNAseq data were assessed for quality using FastQC version 0.11.5 (https://www.bioinformatics.babraham.ac.uk/projects/fastqc/ accessed on 1 January 2020). Trimming was performed with bbduk (sourceforge.net/projects/bbmap/) with a simple clip threshold of ten, average quality threshold of ten, and a minimum read length of 75. Quality was then revisualized with FastQC after trimming and exceeded the phred threshold of 28 needed to be considered high quality sequencing data. Transcriptome assembly and read count quantification were completed with Salmon version 0.11.3 [41] using the Ensembl GRCh38.p7 reference genome [42]. Transcripts per million (TPM) quantification at the transcript level was provided by the Salmon software. Total GILT expression was calculated as the sum of the TPM expression values for the three GILT transcripts (gene name: IFI30): ENST00000407280.4, ENST00000597802.2, and ENST00000600463.1.

### 2.3. Patients, Specimens, and Clinical Dataset

The clinical dataset was comprised of metastatic melanoma patients with either advanced stage III disease with resectable regional metastases or stage IV with distant metastases at the time of study enrollment. Only patients who had not received prior treatment with ICI were included, as prior treatment for melanoma could alter their response to ICI. The final inclusion criteria for the clinical dataset included metastatic melanoma patients with formalin-fixed, paraffin-embedded (FFPE) biopsies of metastatic lesions performed for medical purposes between 2011 and 2016. The hematoxylin and eosin (H&E) stained, as well as unstained, sections were provided by Banner Health and Sonora Quest for this study.

There were *n* = 65 patients that met the inclusion criteria for the study; however, the FFPE specimens were unable to be retrieved for *n* = 19 patients and the FFPE specimens did not contain residual tumor for analysis in *n* = 3 patients. Thus, the final metastatic melanoma clinical dataset was comprised of *n* = 43 patients. Patients’ electronic medical records were utilized to retrieve clinical and pathological data following the institutionally approved protocol (Banner Health; IRB project number: 14-16-0013). The clinical dataset included the following information: subject ID, sex, date of birth, date of metastatic melanoma specimen collection, stage at time of specimen collection, expression of BRAF1, use of BRAF-targeting therapies, use of chemotherapy, use of ICI, and date of last follow-up or date of death. Patients’ survival was updated in March 2021 by first reviewing the electronic medical records for death records. Patients not marked as deceased in the electronic medical records were searched for in the public death records and the Arizona Department of Health Services’ Vital Records Database, which contains the birth and death records of Arizona citizens. For the remaining patients, a confirmed visit at Banner Health after January 2021 was used as the last date of follow-up. The patients who had not been seen at Banner Health after January 2021 were contacted, and the date of contact was used as the last date of follow-up.

### 2.4. Immunohistochemistry

Automated immunohistochemical staining was performed on a Discovery XT Immunostainer (Ventana Medical Systems; Tucson, AZ, USA). Heat-induced epitope retrieval was performed using Discovery Cell Conditioning-1 solution (Ventana Medical Systems). Sections were washed with Reaction Buffer (Ventana Medical Systems) and blocked with BLOXALL (Vector Laboratories, Burlingame, CA, USA) to eliminate endogenous alkaline phosphatase and alkaline phosphatase activity. Sections were stained with rabbit anti-GILT monoclonal antibody (6.67 μg/mL, Spring Bioscience, Pleasanton, CA, USA) diluted in Antibody Diluent with Casein (Ventana Medical Systems), followed by blocking with Antibody Diluent with Casein, and detection with the secondary antibody, UltraMap Red Anti-Rabbit Alkaline Phosphatase (Ventana Medical Systems), and chromagen, DISCOVERY ChromoMap Red Kit (Ventana Medical Systems). A red chromagen was used to differentiate staining from brown melanin pigment. Then, the sections were stained with Hematoxylin II counterstain and Bluing reagent (Ventana Medical Systems). Staining with Confirm Negative Control Rabbit Ig (Ventana Medical Systems), in place of the anti-GILT antibody, served as a negative control. No staining was detected in any specimen with the negative control antibody.

Melanoma cells and tumor-infiltrating APCs were identified based on morphological and histological characteristics, as described [30,31]. Briefly, melanoma cells were identified in H&E-stained sections on the basis of cytomorphologic features. Tumor-infiltrating APCs were identified as single cells with a dendritic morphology scattered among the melanoma cells. The APCs were inconspicuous on the H&E-stained sections, but were identified by their dendritic appearance on the GILT-stained sections. GILT expression within melanoma cells was scored for overall staining (positive or negative), frequency, and intensity of GILT. The frequency of staining was scored quantitatively by taking the area of the melanoma cells that stained positively for GILT over the total area of melanoma cells for all sections of each specimen. Staining intensity was scored categorically as absent (no staining), faint (blush with no vesicular staining), intermediate (vesicular pattern, as found in B cells), or intense (confluent staining, as found in dendritic cells). Staining of each section was scored by a board-certified dermatopathologist (D.J.D.) and dermatologist (K.T.H.) who came to agreement on each case.

### 2.5. Survival and Statistical Analysis

For the RNAseq data, survival analysis was performed with the TPM expression data. The TPM expression values were log_10_ transformed to adjust for the skewness of the expression and a constant of 0.1 was added to each expression value to avoid taking the log of zero. The relationship of GILT mRNA expression and survival was assessed using a Cox proportional hazards model for the combined Liu, Van Allen, and Hugo cohorts. GILT expression was treated as a categorical variable with the two categories being defined by a median split of GILT expression. The relationship of high and low GILT expression to progression-free and overall survival provided by the dataset was assessed using the Cox proportional hazards model from R statistical software version 4.1.0 (R Core Team, Vienna, Austria). The Hugo dataset only provided data for overall survival and was, therefore, excluded from the analysis of progression-free survival. Since there was no significant difference in GILT expression between females and males (Supplementary Figure S1a), sex was not included as a covariate in the Cox proportional hazards model. Age was not provided for the Liu dataset and was therefore not included in the final Cox proportional hazards model. Assessment in the Van Allen cohort alone demonstrated a less than 10% change in the hazard ratio (HR) for GILT with the inclusion of age, indicating that age was not a confounding variable. Multivariate analysis was performed to assess the impact of GILT on survival with adjustments for dataset (Liu vs. Van Allen vs. Hugo) and stage. The addition of stage resulted in a less than 10% change in the HR for the association of GILT with survival. The HR for GILT expression with both overall and progression-free survival changed less than 10% with the addition of the dataset, indicating that the dataset did not confound the association of GILT with survival. A sensitivity analysis was performed with a Cox proportional hazards analysis of GILT expression split into four quartiles; this model was also adjusted by the dataset and stage. The Cox proportional hazards analysis assesses the risk of death per interval of time relative to a baseline risk.

To determine the sample size for the clinical dataset, the simplified assumption that comparison of survival would be performed using a log-rank test (no adjustment for clinical confounders) was made. Additionally, it was assumed that 40% of patients would not have GILT protein expression in metastatic melanoma cells, based on our prior study [31], and that patients without GILT protein expression in metastatic melanoma cells would have a five-year survival of 20%. The clinical study was designed with a sample size of 60 total patients to detect a difference in five-year survival from 20% in those without GILT protein expression versus 50% in those with GILT expression with 80% statistical power, assuming a one-sided log-rank test at the alpha = 0.05 level. Our power was reduced since the available sample size was restricted to 43 patients, and the observed difference in the five-year survival was smaller (27% in those without GILT protein expression versus 43% in those with GILT protein expression).

The association of GILT protein expression in melanoma cells with overall survival was evaluated in all of the patients in the clinical dataset and the subset of patients treated with ICI. The time from the date of metastatic melanoma specimen collection to the date of last follow-up or date of death was used to calculate overall survival. For overall survival analyses, the percentage of GILT-expressing melanoma cells by area was evaluated as a continuous variable. A constant of 0.1183, the smallest non-zero GILT protein expression percentage, was added to each GILT protein expression percentage to avoid taking the log of zero, and then the values were log_10_ transformed, because of the skewness of the GILT protein expression values. Overall survival was evaluated using a Cox proportional hazards model with adjustments for age, sex, and stage at the time of specimen collection in the model, using R software version 4.0.0. The effect of sex in GILT protein expression in melanoma cells was determined, using Wilcoxon rank-sum tests, for all patients in the clinical dataset and the subset of patients treated with ICI. Sex was included as an adjustment in the model because the percentage of GILT-expressing melanoma cells by area was significantly higher in males compared to females when evaluating all of the patients in the clinical dataset (Supplemental Figure S1b) and the subset of patients treated with ICI (Supplemental Figure S1c). In our previously published work, GILT-expressing melanoma cells by area in metastatic melanoma specimens did not differ by sex [31]. Additionally, a greater than 10% change in the HR for GILT was observed with the inclusion of both age and sex in the analysis of the subset of patients in the clinical dataset treated with ICI, suggesting that age and sex were confounding variables. Age and sex were not identified as confounding variables in the analysis for all of the patients in the clinical dataset. However, age and sex were adjusted for in the model to evaluate overall survival in all of the patients in the clinical dataset because age was included in our dataset; there were significant differences in GILT expression between the sexes; and these variables were confounding variables in the subset of patients treated with ICI. Stage at the time of specimen collection was included as an adjustment in the model for all patients in the clinical dataset and the subset of patients treated with ICI. The one patient with stage II melanoma at the time of specimen collection was grouped with the patients with stage III melanoma at the time of specimen collection for analysis. This patient met the inclusion criteria, because the patient was stage IV at the time of study enrollment. Stage was not a confounding variable for all patients in the clinical dataset or the subset of patients treated with ICI. The descriptive characteristics of GILT-expressing melanoma tumor cells were evaluated using Prism 9 software (GraphPad, La Jolla, CA, USA).

## 3. Results

### 3.1. High GILT mRNA Expression Is Associated with Improved Survival in Melanoma Patients Treated with ICI

**Figure 1:**
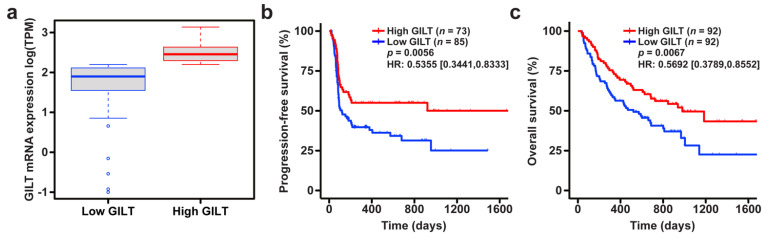
High GILT mRNA expression is associated with improved survival in metastatic melanoma patients treated with immune checkpoint inhibition. (**a**) Distribution of GILT mRNA expression in the high and low GILT categories, split by the median value, in the combined Liu, Van Allen, and Hugo datasets. The bold line in the box plot indicates the median; the upper and lower limits of the boxes indicate the 75th and 25th percentiles, respectively. The lower and upper whiskers indicate the minimum and maximum. Dots outside of the box and whiskers indicate outliers. Association of high and low GILT expression with (**b**) progression-free survival in the combined Liu and Van Allen datasets and (**c**) overall survival in the combined Liu, Van Allen, and Hugo datasets as calculated by a Cox proportional hazards model adjusted for stage and dataset. HR, hazard ratio with 95% confidence interval, indicated in brackets.

To investigate the clinical significance of GILT expression in metastatic melanoma specimens in response to ICI, we determined the association of GILT mRNA expression with overall survival in the combined cohort of patients from the Liu, Van Allen, and Hugo datasets. The association of GILT mRNA expression with progression-free survival was assessed in the combined Liu and Van Allen datasets, since progression-free survival was not available for the Hugo dataset. A range of GILT mRNA expression was observed in melanoma patients from the Liu, Van Allen, and Hugo datasets (Figure 1a), as previously reported in other melanoma patient cohorts [27]. As stage is a prognostic factor for survival, stage was included as a covariate in the survival analysis. The association of GILT with progression-free survival and overall survival remained statistically significant after adjusting for stage, and thus, the effect of GILT is independent of stage. Patients in the Liu and Hugo datasets were treated with anti-PD-1, and patients in the Van Allen dataset were treated with anti-CTLA-4 (Table 1). To adjust for the effect of dataset, which includes differences in therapy, dataset was included as a covariate in the survival analysis. The final multivariate model, including categorical GILT expression, stage, and dataset, demonstrated a significant association of high GILT mRNA expression with improved progression-free and overall survival in patients treated with ICI (Figure 1b,c). Median progression-free survival in the Liu and Van Allen datasets was 120 days for the low GILT expression group and not reached for the high GILT expression group. The median overall survival for the combined Liu, Van Allen, and Hugo datasets was 521 and 980 days for the low and high GILT expression groups, respectively. A sensitivity analysis, where GILT expression was divided into four quartiles, suggested that quartiles one and two had similar survival, which significantly differed from the survival for quartiles three and four (data not shown). These data demonstrate an association of high GILT mRNA expression with improved survival in metastatic melanoma patients treated with ICI.

### 3.2. Variable GILT Protein Expression in Melanoma Cells of Metastatic Tumor Specimens

**Figure 2:**
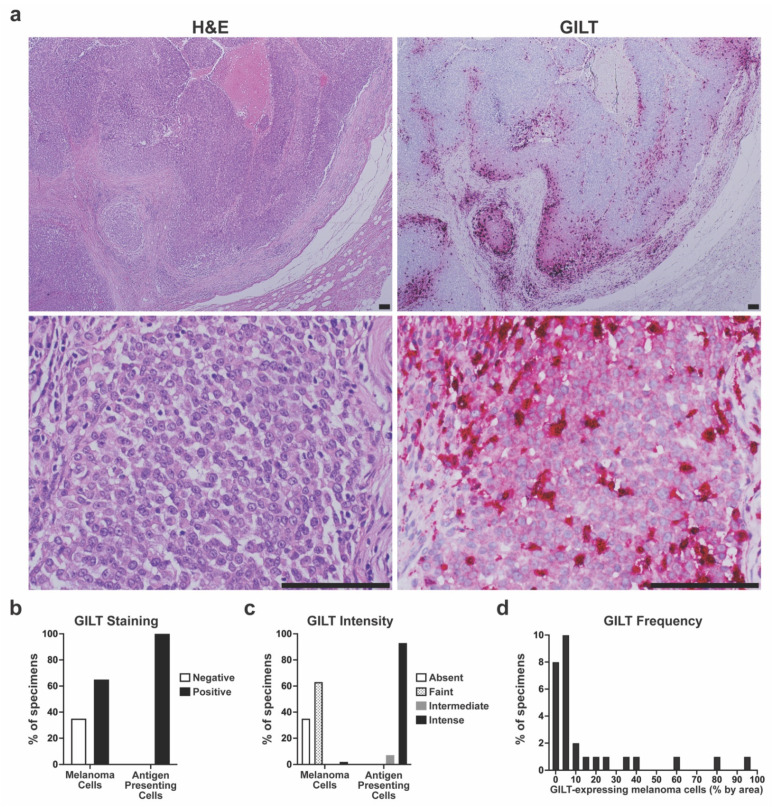
Variable GILT protein expression in melanoma cells of metastatic tumor specimens. (**a**) Representative hematoxylin and eosin (H&E) staining (left panels) and GILT immunohistochemical (IHC) staining of serial sections of metastatic melanoma specimens. Lower magnification (top panels) illustrates GILT staining in a subset of melanoma cells. Higher magnification (bottom panels) illustrates faint GILT staining within melanoma cells and intense GILT staining within antigen presenting cells with a dendritic morphology. Bar = 100 µm. (**b**) Percentage of metastatic melanoma specimens with negative or positive GILT staining in melanoma cells and antigen presenting cells. (**c**) Intensity of GILT staining within melanoma cells and antigen presenting cells in metastatic melanoma specimens. (**d**) Distribution of GILT-expressing melanoma cells within the *n* = 28 specimens with positive GILT staining in melanoma cells.

As high GILT mRNA expression was associated with improved survival in metastatic melanoma patients treated with ICI, we sought to study the significance of GILT protein expression in metastatic melanoma specimens. To evaluate GILT protein expression in metastatic melanoma, a clinical dataset of metastatic melanoma specimens was generated and annotated with clinical information. The demographics of the *n* = 43 patients included in the clinical dataset are detailed in Table 2. FFPE regional or distant metastatic specimens for each patient were stained using H&E (Figure 2a left panels) and for GILT using IHC (Figure 2a right panels). Figure 2b shows that 65% of the patient specimens (28/43) had positive GILT staining within melanoma cells, and all 43 patient specimens had GILT-stained tumor-infiltrating APCs. Variable GILT protein expression within melanoma cells is consistent with a previous study, where GILT protein expression in melanoma cells was identified in 58% of cutaneous metastatic melanoma specimens [31]. The staining intensity of GILT within melanoma cells ranged from absent to intense with most specimens exhibiting faint GILT staining within melanoma cells (Figure 2c). Within APCs, the GILT staining intensity was either intermediate or intense, with nearly all of the specimens having intense GILT staining within APCs. In evaluating all of the patients in the clinical dataset, the mean and median frequency of GILT-expressing melanoma cells was 9.9% and 0.9%, respectively. Within the *n* = 28 patients with GILT-expressing melanoma cells, the mean GILT expression was 15.2% and the median was 4.5% (Figure 2d). These data support that GILT-expressing APCs are uniformly present within metastatic melanoma and that GILT protein expression varies within metastatic melanoma cells of the same tumor and between patients.

### 3.3. High GILT Protein Expression in Melanoma Cells Is Associated with Improved Overall Survival in Metastatic Melanoma Patients Treated with ICI

**Figure 3:**
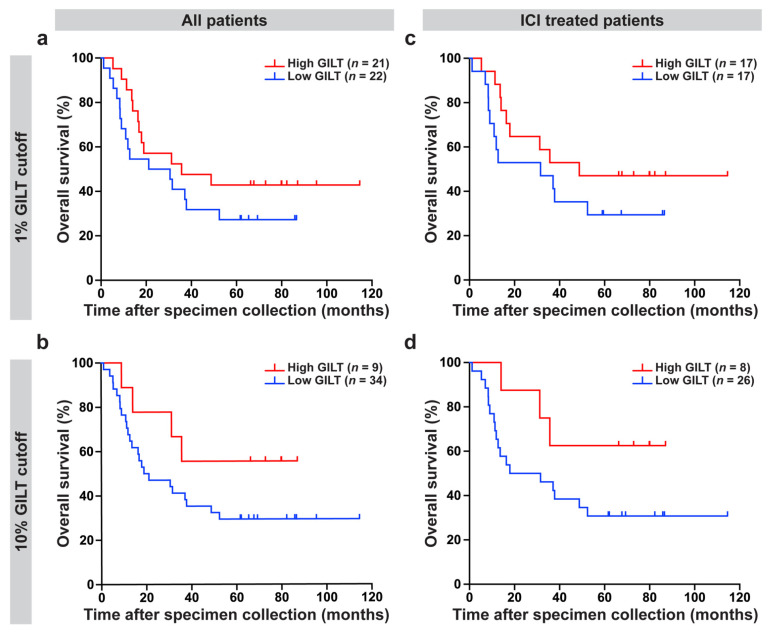
High GILT protein expression in melanoma cells is associated with improved overall survival in metastatic melanoma patients treated with immune checkpoint inhibitors (ICI). For the overall survival analyses, GILT protein expression in melanoma cells was evaluated as a continuous variable. The relationship between overall survival and GILT protein expression in melanoma cells was assessed using the Cox proportional hazards model, with stage at the time of specimen collection, age, and sex included as adjustment factors. (**a**,**b**) High GILT protein expression in melanoma cells was associated with a longer overall survival in all of the patients in the clinical dataset (*p* = 0.1270; HR [95% CI] = 0.7139 [0.4630, 1.1010]); however, it did not reach statistical significance. (**c**,**d**) In the subset of patients treated with ICI, high GILT protein expression in melanoma cells was associated with a significantly improved overall survival (*p* = 0.0347; HR [95% CI] = 0.5682 [0.3363, 0.9601]). Kaplan–Meier plots to visualize data using a cutoff of (**a**,**c**) 1% and (**b**,**d**) 10% of melanoma cells with GILT staining. HR, hazard ratio; CI, confidence interval.

To investigate the clinical significance of GILT protein expression in metastatic melanoma cells, the association of GILT protein expression with overall survival was determined for all of the patients in the clinical dataset and in the subset of patients in the clinical dataset treated with ICI. In examining all of the patients in the clinical dataset, high GILT protein expression was associated with a longer overall survival (Cox proportional hazards model, *p* = 0.1270; HR [95% CI] = 0.7139 [0.4630, 1.1010]) adjusted for stage, age, and sex; however, it did not reach statistical significance. To visualize these data, Kaplan–Meier plots of the patients in the clinical dataset were separated into high or low GILT protein expression groups, using cutoffs of 1% (same groupings as median) and 10% of the melanoma cells expressing GILT (Figure 3a,b) were generated. Using the 1% cutoff, the low and high GILT expression groups had a median survival of 26 months and 36 months, respectively (Figure 3a). With a 10% cutoff, the median survival was 20 months in the low GILT expression group and was not reached in the high GILT expression group (Figure 3b).

As high GILT mRNA expression was associated with improved survival in metastatic melanoma patients treated with ICI and as GILT has a well-established role in antigen presentation and enhancing T cell responses [23,24,25,43], the association of GILT protein expression in metastatic melanoma cells with clinical outcome in patients treated with ICI was tested. High GILT protein expression in melanoma cells was associated with significantly improved overall survival in the *n* = 34 patients treated with ICI (Cox proportional hazards model, *p* = 0.0347; HR [95% CI] = 0.5682 [0.3363, 0.9601]) adjusted for stage, age and sex. Kaplan–Meier plots for the patients in the clinical dataset treated with ICI using cutoffs of 1% and 10% of melanoma cells expressing GILT (Figure 3c,d) were generated to visualize the data. In the subset of ICI-treated metastatic melanoma patients, using the 1% cutoff, patients with low and high GILT expression in melanoma cells had a median survival of 32 months and 49 months, respectively (Figure 3c). Using a 10% cutoff, the median survival was 25 months in the low GILT expression group and was not reached in the high GILT expression group (Figure 3d). These data support that high GILT protein expression in melanoma is associated with improved overall survival in metastatic melanoma patients treated with ICI.

## 4. Discussion

We demonstrate an association of high GILT mRNA expression in metastatic melanoma specimens with improved progression-free and overall survival in patients treated with ICI. To investigate in which cell type GILT expression is associated with improved survival, we generated a new dataset of metastatic melanoma specimens annotated with clinical data. While GILT protein expression in melanoma cells was variable, with 65% of specimens containing GILT-expressing melanoma cells and a median of 1% of the melanoma cells staining for GILT, all specimens contained GILT-expressing APCs. High GILT protein expression in melanoma cells was associated with improved overall survival in patients treated with ICI. The association of GILT mRNA and protein expression with survival was independent of stage. Together, these data support further investigation into GILT as a predictive biomarker of response to ICI.

GILT expression as a biomarker of improved ICI response in melanoma patients is supported by prior studies. GILT’s most well-described biological function is in the MHC class II antigen presentation pathway [22,44,45]. Using RNAseq data from bulk tumor specimens, high GILT expression, high MHC class II expression, and an active and intact MHC class II antigen presentation pathway are each associated with improved survival in melanoma patients before the advent of ICI [27,46]. Proteomic evaluation of metastatic melanoma specimens revealed increased expression of MHC class I and II antigen presentation pathway members, including GILT, in patients who responded to anti-PD-1 and in patients who responded to adoptive cell therapy [47]. A limitation of studies using RNAseq and proteomic data from bulk tumor specimens is that the cell type expressing the gene or protein of interest is not identified. Since the majority of MHC class II expression in RNAseq data from melanoma samples is derived from APCs [48] and tumor-infiltrating APCs are generally GILT and MHC class II positive (current study and [31]), there is a need to assess GILT and other MHC class II pathway components in specific cell types, such as tumor cells, as performed in the current study.

Consistent with our finding of the association of GILT expression in melanoma cells with improved survival in ICI-treated patients, other groups have reported an association of MHC class II expression on melanoma cells with improved response to ICI. Johnson et al. reported the association of MHC class II expression on greater than 5% of melanoma cells with improved response to anti-PD-1/PD-L1 [49]. Rodig et al. demonstrated that MHC class II expression on greater than 1% of melanoma cells was associated with improved response to anti-PD-1 [7]. The association of both GILT and MHC class II expression in melanoma cells with improved response to ICI supports future investigation into a causal role of GILT in MHC class II presentation on melanoma cells in the response to ICI. Alternatively, GILT expression may be indirectly associated with improved response to ICI or may be associated with improved survival due to factors other than response to ICI.

Studies in murine cancer models support a role for MHC class II-restricted antigen presentation in tumor cells enhancing anti-tumor immune responses. Murine tumors expressing MHC class II exhibit enhanced rejection and/or reduced in vivo tumor growth compared to MHC class II-deficient tumors in breast cancer [50,51,52,53], sarcoma [54,55], colon cancer, renal carcinoma [55], and T cell lymphoma [56]. In breast cancer cells, MHC class II expression confers response to ICI, using either combination therapy with anti-PD-1 and anti-Lag-3 or treatment with anti-CTLA-4 [52,53]. Expression of MHC class II, but not MHC class I, confers responsiveness to anti-PD-1 therapy in colon cancer and B and T cell lymphoma models [56] via cytotoxic CD4 T cells. In colon cancer, sarcoma [55], and breast cancer models [50,51,55], MHC class II-mediated tumor rejection is dependent on both CD4 and CD8 T cells. MHC class II-expressing melanoma cells and breast cancer cells are capable of MHC class II-restricted processing of protein antigens and presentation of peptide epitopes to CD4 T cells [51,57,58,59,60,61,62]. Although tumor rejection and response to ICI can occur in the absence of MHC class II expression on tumor cells in some models [18,21], MHC class II expression on tumor cells has the potential to enhance anti-tumor immunity. Thus, mechanistic studies demonstrate a role for MHC class II-restricted antigen presentation in the anti-tumor immune response and support a causal role for MHC class II-restricted presentation by tumor cells in the anti-tumor immune response.

GILT expression in melanoma cells is anticipated to improve anti-tumor immunity through improved MHC class II-restricted presentation in the tumor microenvironment. GILT’s reductase activity facilitates the presentation of certain epitopes from disulfide-bond containing proteins, which are predicted to be buried within the tertiary structure [23,25,43,44,45]. In the absence of GILT, there is diminished presentation of GILT-dependent epitopes and enhanced presentation of peptides, which are anticipated to be readily accessible for MHC class II binding without protein disulfide bond reduction [23,25,43,44,45,63]. Induction of GILT expression in melanoma cells alters the peptide repertoire presented by MHC class II in melanoma cells [24,26]. While APCs, in general, constitutively express GILT, melanoma cells have variable GILT expression. GILT expression in melanoma cells is anticipated to promote anti-tumor immunity by facilitating the presentation of MHC class II-restricted peptides by tumor cells in the microenvironment, which are the same set of epitopes presented by APCs and involved in priming CD4 T cell responses.

## 5. Conclusions

In summary, high GILT mRNA expression in metastatic melanoma specimens was associated with improved progression-free and overall survival in patients treated with ICI, using RNAseq datasets. There was variable GILT protein expression in malignant melanocytes in metastatic melanoma specimens, with 65% of specimens containing GILT-expressing melanoma cells. In patients treated with ICI, high GILT protein expression within melanoma cells was associated with improved overall survival. These studies support that high GILT mRNA expression in bulk tumor samples and high GILT protein expression in melanoma cells are associated with improved survival in ICI-treated melanoma patients. The association of GILT mRNA and protein expression with survival was independent of cancer stage. These findings support further investigation into GILT as a biomarker to predict which patients will respond to ICI, with the potential to improve patient care, reduce healthcare costs, and facilitate appropriate selection of therapies for patients with metastatic melanoma.

## Figures and Tables

**Table 1:** Demographics of metastatic melanoma patients from the Liu, Van Allen, and Hugo datasets of RNAseq data.

Liu Dataset	
Number of patients	*n* = 122
Females	*n* = 51
Males	*n* = 71
Length of follow up (mean ± SD)	18.8 ± 12.8 months
Melanoma subtype	
Cutaneous	73% (89/122)
Occult	16% (19/122)
Acral	7% (8/122)
Mucosal	5% (6/122)
Stage	
M0	8% (10/122)
M1a	6% (7/122)
M1b	11% (14/122)
M1c	76% (91/122)
Immune checkpoint inhibitors	
Anti-PD-1 therapy alone	100% (122/122)
Other treatments	
MAPK therapy prior to anti-PD-1 therapy	15% (18/122)
MAPK therapy after anti-PD-1 therapy	9% (11/122)
Anti-CTLA-4 therapy prior to anti-PD-1 therapy	39% (48/122)
Anti-CTLA-4 therapy after anti-PD-1 therapy	9% (11/122)
Combined anti-CTLA-4/anti-PD-1 after anti-PD-1 therapy	13% (16/122)
Van Allen Dataset	
Number of patients	*n* = 36
Females	*n* = 9
Males	*n* = 27
Age of patients at diagnosis (mean ± SD)	60.3 ± 15.5 years
Length of follow up (mean ± SD)	18.5 ± 15.5 months
Melanoma subtype	
Cutaneous	100% (36/36)
Stage	
M0	3% (1/36)
M1a	6% (2/36)
M1b	17% (6/36)
M1c	75% (27/36)
Immune checkpoint inhibitors	
Anti-CTLA-4 therapy alone	100% (36/36)
Other treatments	
BRAF inhibitor prior to anti-CTLA-4 therapy	11% (4/36)
BRAF inhibitor after anti-CTLA-4 therapy	33% (6/36)
Hugo Dataset	
Number of patients	*n* = 26
Females	*n* = 8
Males	*n* = 18
Age of patients at diagnosis (mean ± SD)	59.3 ± 15.1 years
Length of follow up (mean ± SD)	17.3 ± 10.4 months
Melanoma subtype	Not reported
Stage	
M0	4% (1/27)
M1a	7% (2/27)
M1b	11% (3/27)
M1c	78% (21/27)
Other treatments	
MAPK therapy prior to anti-PD-1 therapy	15% (12/27)

**Table 2:** Demographics of patients in the metastatic melanoma clinical dataset.

Metastatic Melanoma Specimen Dataset	
Number of patients	*n* = 43
Females	*n* = 11
Males	*n* = 32
Average age of all patients at time of collection (mean ± SD)	64.1 ± 14.3 years
Average length of follow up (mean ± SD)	39.6 ± 31.9 months
Stage at the time of specimen collection	
II	2% (1/43)
III	28% (12/43)
IV	70% (30/43)
BRAF V600E status	
Positive	35% (15/43)
Negative	56% (24/43)
Not tested	9% (4/43)
Immune checkpoint inhibitors	
Anti-CTLA-4 therapy alone	35% (15/43)
Anti-PD-1 therapy alone	30% (13/43)
Anti-CTLA-4 and anti-PD-1 therapy	14% (6/43)
Other treatments	
BRAF inhibitor therapy	21% (9/43)
Chemotherapy	33% (14/43)

## Data Availability

Publicly available data was used for this study. Both the Liu and Van Allen datasets presented in this study are available in dbGaP with the following accession number: phs000452.v3.p1. RNAseq data were obtained for the Hugo et al. dataset from the gene expression omnibus repository with accession number GSE78220.

## Supplementary Materials

The following are available online at https://www.mdpi.com/article/10.3390/cancers14092200/s1, Figure S1: No difference in GILT mRNA expression in melanoma specimens between females and males in the combined Liu and Van Allen datasets; however, in the clinical dataset there was a higher percentage of GILT protein expressing melanoma cells in males.
