# Supplementary material for: High GILT Expression Is Associated with Improved Survival in Metastatic Melanoma Patients Treated with Immune Checkpoint Inhibition
Source: Cancers (Basel). 2022 Apr 28;14(9):2200. doi: 10.3390/cancers14092200 (PMC9100272; doi:10.3390/cancers14092200)
Supplement: Supplementary file 1 [file cancers-14-02200-s001.zip › cancers-1664091-supplementary.pdf]

cancers

MDPI

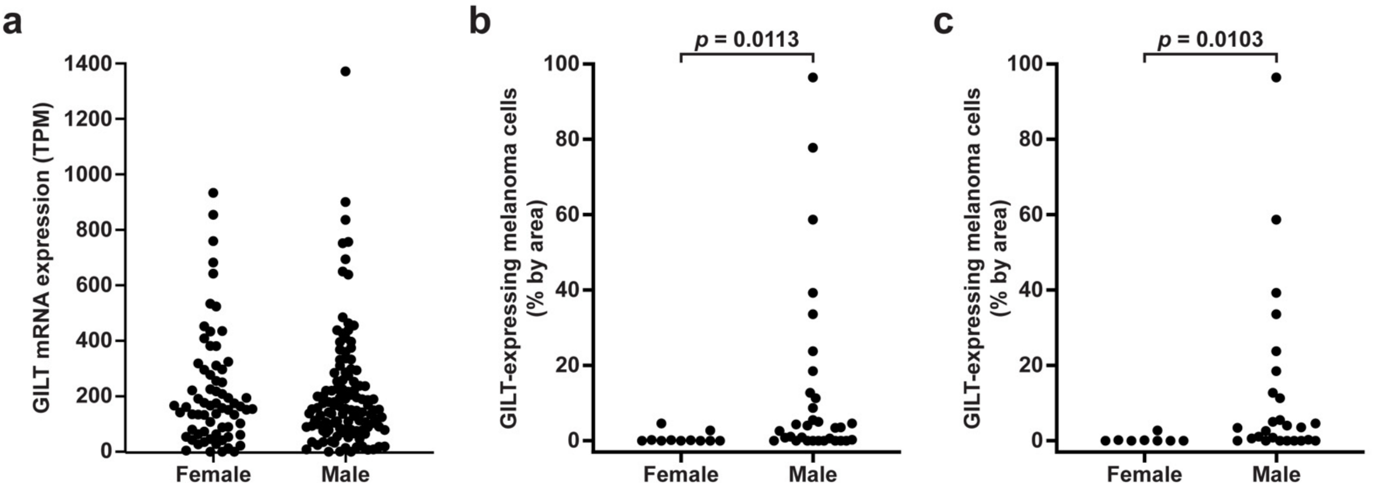

Supplemental Figure S1. No difference in GILT mRNA expression in melanoma specimens between females and males in the combined Liu and Van Allen datasets; however, in the clinical dataset there was a higher percentage of GILT protein expressing melanoma cells in males. (a) No statistically significant difference in GILT mRNA expression between males and females in bulk tumor samples in the combined Liu and Van Allen datasets (Wilcoxon rank-sum test, $p > 0.9999$). Distribution of the percent of GILT-expressing melanoma cells by area in females and males in the metastatic melanoma clinical dataset in (b) all patients and (c) the subset of patients treated with immune checkpoint inhibition (ICI). Wilcoxon rank-sum tests shows males have a higher percentage of melanoma cells expressing GILT protein compared to females, in comparisons of all the patients in the clinical dataset and the subset of patients treated with ICI. Dots represent each patient value.
